# The Misconceptions and Determinants of Diabetes Knowledge in Patients with Diabetes in Taiwan

**DOI:** 10.1155/2020/2953521

**Published:** 2020-06-18

**Authors:** Chia-Chia Chen, Chien-Lung Chen, Yu Ko

**Affiliations:** ^1^Department of Pharmacy, College of Pharmacy, Taipei Medical University, Taipei, Taiwan; ^2^Department of Pharmacy, Landseed International Hospital, Taoyuan, Taiwan; ^3^Division of Nephrology, Landseed International Hospital, Taoyuan, Taiwan; ^4^Research Center for Pharmacoeconomics, College of Pharmacy, Taipei Medical University, Taipei, Taiwan

## Abstract

**Aim:**

This study aimed at (1) examining the misconceptions of patients with diabetes in Taiwan and (2) examining the association between patients' diabetes knowledge and their demographic characteristics.

**Methods:**

A cross-sectional survey was conducted at a metropolitan hospital in northern Taiwan. A total of 501 outpatients with diabetes were recruited, mostly from the hospital's endocrinology and metabolism clinic, nephrology clinic, and dialysis center. A self-developed questionnaire that consisted of demographic information and a diabetes knowledge test was administered. The knowledge test included 10 True/False questions and 6 multiple-choice questions that aimed at identifying patients' common misconceptions about diabetes.

**Results:**

A perfect score on the diabetes knowledge test was 16 points, and the mean ± SD score of the respondents was 11.5 ± 2.8. The most common misconception was “People can always feel when their blood sugar level is high.” (64%), followed by “Taking insulin hurts the kidneys and may result in a need for dialysis.” (52%) and “Being a vegetarian helps control blood sugar levels.” (48%). The total knowledge scores were significantly associated with education levels (*r*_s_ = 0.39, *p* < 0.001), average monthly income (*r*_s_ = 0.28, *p* < 0.001), and age (*r*_s_ = −0.34, *p* < 0.001).

**Conclusions:**

Certain misconceptions are prevalent among patients with diabetes, particularly in those with older age, lower education levels, or lower income. Healthcare providers need to work to eliminate common misconceptions and modify diabetes educational programs accordingly to help patients manage diabetes more effectively.

## 1. Introduction

Globally, there are more than 400 million people affected by diabetes, and its prevalence has continuously increased. The International Diabetes Federation (IDF) has projected that diabetes will influence 693 million in 2045 [1]. Moreover, the World Health Organization (WHO) estimated that 1.5 million people directly died from diabetes and 2.2 million died from diabetes-related complications [2]. In Taiwan, there are two million people suffering from diabetes, which accounts for approximately one-tenth of the population [3]. In addition, the expenditure for and mortality of diabetes are ranked as second and fifth place among diseases in Taiwan, respectively [3, 4].

To prevent or delay the progression of diabetes and related complications, medications and lifestyle management are both important [5], and patients play an essential role in the treatment of their own diseases [6]. It is important for patients to have adequate and correct information about their medications, diet, and lifestyle management, and misconceptions can be a barrier to control diabetes. Indeed, lack of awareness and insufficient understanding of diabetes could lead to adverse consequences. In recent years, a few studies have investigated patients' knowledge about their diabetes [7–10] or examined the association between diabetes-related self-care knowledge and glycemic control [10–13]. For example, a study conducted by Mann et al. demonstrated that certain misconceptions about diabetes and its treatment were associated with poor glycemic control [10]. Moreover, a knowledge, attitude, and practice (KAP) study demonstrated that patients' knowledge scores positively correlated with their glycemic control [13]. Furthermore, a recent qualitative study [14] found that patients who highly adhered to their antidiabetic medications may still have insufficient knowledge about their daily self-management practices.

Misconceptions about diseases and treatments can vary across cultures and populations. In order to enhance and improve patient education on diabetes, a better understanding of misconceptions and associated factors in our local population is important. Therefore, this study aimed at (1) examining the misconceptions of patients with diabetes in Taiwan and (2) examining the association between patients' diabetes knowledge and their demographic characteristics. We aimed at identifying knowledge gaps and associated factors in order to help healthcare providers and educators tailor their diabetes education and improve diabetes management.

## 2. Methods

### 2.1. Subjects

We conducted a cross-sectional survey to recruit a convenience sample of outpatients with diabetes at a metropolitan hospital, mostly in the hospital's endocrinology and metabolism clinic, nephrology clinic, and dialysis center. The study hospital provides healthcare services for people in Taoyuan City, located in northern Taiwan. It has 630 beds and serves over 2,500 outpatients per day. The inclusion criteria were (1) a confirmed diagnosis of diabetes and (2) age 20 years or older. The exclusion criteria were (1) being pregnant or (2) cognitive impairment or inability to communicate with the survey administrators.

### 2.2. Survey Instrument

This study sought to assess patients' knowledge of diabetes using a questionnaire specifically developed for this study based on literature review, [7, 8, 10, 15–24] paper- and web-based patient education materials, and expert opinion. During questionnaire design, first, the questions collected from the above sources were divided into categories such as efficacy, side effects, medication use, complications, daily healthcare, and disease awareness. Then, questions that were considered appropriate and relevant for our patients were selected from each category to form a draft of the questionnaire, which was further modified by an expert panel that included a physician who was the head of the endocrine and metabolism business unit and a senior pharmacist with survey research and diabetes counselling experience. Moreover, to enhance the questionnaire's readability and clarity, a pilot test of 25 eligible patients was conducted at the start of the survey. No change was deemed necessary based on the comments of the pilot testees.

The final version of the study questionnaire comprised two sections: demographic information and a diabetes knowledge test. The demographic information included patients' age, gender, weight, height, level of education, average monthly income, type of treatments, and the number of years they had lived with diabetes. The knowledge test consisted of 10 True/False questions and 6 multiple-choice questions that aimed to identify patients' common misconceptions about the disease. For instance, “Being a vegetarian helps control blood sugar levels” and “Skipping breakfast helps control blood sugar levels” represented the myths of blood sugar control. “Taking insulin hurts the kidneys and may result in a need for dialysis” probed concerns with insulin treatment. “People can always feel when their blood sugar level is high” assessed patients' propensity to diet or take medications as a result of sensory rather than objective information (e.g., blood glucose finger stick). Moreover, multiple-choice questions such as “What can poor diabetes management lead to?” examined patients' understanding of the consequences of poor blood sugar control while “Which of the following is good for diabetes control?” investigated patients' awareness of a healthy life style. To avoid guessing, a response option of “I am not sure” was provided for all knowledge questions.

### 2.3. Procedures

Our cross-sectional survey was conducted from February 2018 to May 2018. The study protocol and the final version of the study questionnaire were approved by the institutional review board of the study hospital (LSHIRB No. 17-038-B1). Informed consent was obtained from all participants. The questionnaire was administered by two well-trained interviewers and took 10 to 15 minutes to complete. To facilitate the interview process and enhance patient comprehension, supplementary materials such as a simplified research protocol and written layman explanations of medical terms were provided. In addition, patients' hospital medical records were retrieved to extract their most recent HbA1c value.

### 2.4. Statistical Analysis

Data were analyzed using SPSS software version 19 (IBM SPSS Statistics) with a *p* value of < 0.05 considered to be statistically significant. Means and standard deviations were used to describe continuous variables while frequency and percentages were used for categorical variables. Correlations between the knowledge test scores and patient characteristics were examined by Student's *t* test, ANOVA with Scheffe's test, and Spearman's rank correlation test where appropriate.

## 3. Results

A total of 501 completed questionnaires were received. Males accounted for 56.3% of the respondents, and the mean ± SD of age, BMI, duration of diabetes, and HbA1c levels were 60.1 (±13.1) years, 26.9 (±4.4) kg/m^2^, 10.1 (±8.4) years, and 7.9 (±1.8)%, respectively. Nearly one-third of the respondents (*n* = 177, 35.3%) completed high school, and more than half of the respondents (*n* = 276, 55.1%) had no regularly monthly income. In addition, 88.4% of the respondents were taking oral antidiabetic drugs and 28.7% were on insulin. The respondents' characteristics are summarized in Table 1.

A perfect score on the diabetes knowledge test was 16 points, and the mean ± SD score of the respondents was 11.5 ± 2.8. The common misconceptions among respondents are presented in Table 2. The most common misconception was: “people can always feel when their blood sugar level is high,” which was incorrectly perceived as a true statement by almost two-thirds of the respondents. In addition, around half of the respondents shared the misconceptions that “taking insulin hurts the kidneys and may result in a need for dialysis” and “being a vegetarian helps control blood sugar levels”.

The results from Spearman's rank correlation showed that the total knowledge scores were significantly associated with education levels (*r*_s_ = 0.39, *p* < 0.001), average monthly income (*r*_s_ = 0.28, *p* < 0.001), and age (*r*_s_ = −0.34, *p* < 0.001) whereas the total scores were not correlated with gender, BMI, or duration of diabetes. Furthermore, there was a negative association between the test scores and HbA1c, but it did not reach statistical significance (*r*_s_ = −0.07, *p* = 0.10).

## 4. Discussion

The most common misconception found in the present study is that “People can always feel when their blood sugar level is high,” with about 64% of respondents answering that the statement was true. However, unlike hypoglycemia, a high blood sugar level has no early signs or symptoms, and it may develop slowly over a long period of time without awareness. If untreated, it can lead to serious complications [25, 26]. As a result, self-monitoring of blood glucose (SMBG) is recommended for people with diabetes to ensure that their blood sugar level stays within the target range [27]. With SMBG and not patient symptoms being a pillar of good diabetes management, it is important for health professionals to correct this misconception and emphasize the importance of glycemic control regardless of symptoms during patient encounters.

In our study, more than half of the respondents believed that taking insulin would hurt the kidneys and could result in a need for dialysis. In general, diabetes and its related complications progress over time. Many people struggle with their concerns about insulin and delay insulin therapy until their blood sugar control worsens, at which time it is already sometimes too late to delay or prevent kidney damage [22, 28]. Therefore, some patients mistakenly believe that insulin therapy will lead to dialysis when in fact it could prevent it. Another common misconception is “Being a vegetarian helps control blood sugar levels.” It may offer certain benefits by leading to a balanced plant-based diet [29], but there is a lack of consistent evidence to show that a vegetarian diet improves glycemic control [30]. Moreover, a proper vegetarian diet is not equal to a “meat-free” diet. Imbalanced diet can affect nutrient intake and even blood sugar levels. It is thusly important to educate patients to keep a healthy balanced diet and stay within an appropriate energy intake range [30].

We found that the determinants of better diabetes knowledge were higher education levels, younger age, and higher monthly income. Similar findings were reported by other studies in China, India, Kuwait, Jordan, and Costa Rica [13, 15, 17, 20, 31]. The elderly and less-educated patients may have had lower knowledge test scores because of deep-rooted misconceptions about diabetes ingrained years ago or perhaps because of difficulty understanding health education materials or newer information. Health education plays an important role in disease control, particularly for chronic conditions like diabetes [12, 32–34]. Previous studies have shown that there is a need for well-designed diabetes educational programs that could assist with the management of diabetes [35–37]. As healthcare resources and time are limited, it is critical for healthcare providers or decision-makers to decide which patients should be given more attention and what kind of health education should be provided. Moreover, the findings of this study identify the knowledge gaps and common misconceptions of patients with diabetes in Taiwan, in which information will be useful for patient education and counselling.

There are some limitations to the present study. First, the knowledge test was prone to guessing despite the response option of “I am not sure.” Second, as a convenience sample was recruited in an outpatient setting, respondents could be healthier than the general diabetic population. As such, the actual knowledge of the diabetic patients could have been poorer than observed. Also, the questions included in the knowledge test were selected by literature review and expert opinion based on relevance and importance. Due to the concern of respondent burden, not all essential aspects of diabetes knowledge were tested.

## 5. Conclusion

Certain misconceptions are prevalent among patients with diabetes, particularly in those with older age, lower education levels, or lower income. Healthcare providers need to work to eliminate common misconceptions and modify diabetes educational programs accordingly to help patients manage diabetes more effectively.

## Figures and Tables

**(a):** 

Variable	Mean	Standard deviation
Age (years)	60.1	13.1
BMI (kg/m^2^)	26.9	4.4
Duration of diabetes (years)	10.1	8.4
HbA1c (%)	7.9	1.8
Knowledge test score	11.5	2.8

**(b):** 

Variable	*n*	%
Gender		
Male	282	56.3
Female	219	43.7
Level of education		
No formal education	29	5.8
Elementary school or kindergarten	100	20.0
Junior high school	108	21.6
Senior high school/vocational high school/junior college	177	35.3
University	70	14.0
Masters	14	2.8
PhD	3	0.6
Average monthly income (TWD)		
No income	276	55.1
Less than 15,000	21	4.2
15,001-30,000	51	10.2
30,001-45,000	70	14.0
45,001-60,000	48	9.6
60,001-75,000	14	2.8
More than 75,001	21	4.2
Type of treatment		
Lifestyle management	166	33.1
Oral antidiabetic agent	443	88.4
Insulin	144	28.7
Injectable non-insulin drug	7	1.4

**Table 2:** Common misconceptions about diabetes among respondents.

Questions	No. of respondents with a wrong answer	% incorrect
**(False)** People can always feel when their blood sugar level is high.	322	64%
**(False)** Taking insulin hurts the kidneys and may result in a need for dialysis.	261	52%
**(False)** Being a vegetarian helps control blood sugar levels.	241	48%
Foot ulcers are common in people with diabetes. Which of the following activities is not correct for foot care? (i) Check feet every day and keep them clean. (ii) Wear socks and shoes that fit comfortably. **(iii) Use a hot water bag or electric blanket or soak feet in hot water to improve blood circulation to the feet.**	235	47%
**(False)** Women with diabetes should avoid pregnancy or they will pass diabetes to their children.	223	45%
**(False)** Only sugar can affect blood sugar levels. As a result, as long as patients with diabetes stop eating sweets, their diabetes will improve.	174	35%
**(True)** Patients with diabetes do not have to avoid all kinds of sweets.	173	35%
**(True)** Even if people with diabetes are taking blood sugar-lowering medication or insulin, they should not overeat or overdrink.	107	21%
What can poor diabetes management lead to? (i) Kidney problems (nephropathy). (ii) Heart diseases (cardiovascular diseases). **(iii) Both of the above.**	105	21%

Correct answers are shown in boldface.

## Data Availability

The data used to support the findings of this study are available from the corresponding author upon request.
